# Impact of Sample Size and Its Estimation in Medical Research

**DOI:** 10.1016/j.focus.2025.100451

**Published:** 2025-09-27

**Authors:** Wenqi Gan

**Affiliations:** Department of Public Health Sciences, University of Connecticut School of Medicine, Farmington, Connecticut, United States.

**Keywords:** Sample Size, Statistical Power, Confidence Interval, Biostatistics, Epidemiologic Methods, Result Reliability

## Abstract

**Introduction:**

In medical research, study samples are used to estimate and compare characteristics of target populations. However, because samples inevitably differ from their target populations, statistical inferences are subject to inherent uncertainty. Among various contributing factors, sample size plays a critical role in determining the reliability and precision of research findings. This study used data simulations to examine how sample size affects statistical outcomes and its relationships with key metrics of statistical inference.

**Methods:**

Two normal distributions, representing the null and alternative hypotheses, were generated to illustrate the relationships between type I error, type II error, statistical power, and sample size. Statistical power and theoretical *p*-values were calculated using a two-sample *t*-test for equal sample sizes ranging from 10 to 300 per group and Cohen’s standardized effect sizes (*d*) of 0.2, 0.5, and 0.8 to demonstrate the interactions between these factors. Additionally, five representative scenarios were constructed to compare *p*-values and 95% confidence intervals in relation to statistical significance, clinical importance, and precision of effect estimates.

**Results:**

Required sample size is determined by expected effect size, variability, significance level, and desired statistical power. As sample size increases, statistical power increases and *p*-values decrease when a true effect is present; both metrics gradually level off as they approach their theoretical limits. In contrast, effect estimates remain relatively stable, while their 95% confidence intervals become narrower, reflecting improved precision. Compared with *p*-values, confidence intervals provide more comprehensive information, including direction, magnitude, precision, statistical significance, and clinical importance of effect estimates. Therefore, reporting effect estimates and 95% confidence intervals alongside *p*-values is essential for transparent and meaningful interpretation of research findings.

**Conclusions:**

Sample size estimation is a critical component of rigorous study design. It ensures adequate statistical power, improves precision, and facilitates meaningful interpretation of research findings.

## INTRODUCTION

In medical research, study samples are typically used to represent the target populations from which they are drawn, and data from these samples are used to estimate and compare characteristics of target populations.^1^, ^2^, ^3^ However, because samples inevitably differ from their target populations, statistical inferences are subject to inherent uncertainty and imprecision. Multiple factors may contribute to unreliable findings, including sampling variability, measurement error, selection bias, confounding, and flawed study design.^2^, ^3^, ^4^ While these factors are important, this study focuses specifically on the impact of sample size on statistical inference.

Assuming a study sample is representative of the target population and free from major sources of bias and confounding, sample size plays a critical role in determining the reliability and precision of research findings. A sample that is too small may yield inconclusive or potentially misleading results, whereas an excessively large sample may waste resources and detect trivial effects that are statistically significant but clinically negligible.^5^^,^^6^ Therefore, sample size estimation is a critical component of rigorous study design, ensuring that an appropriate number of subjects are enrolled to achieve study objectives with adequate statistical power.^7^

Understanding the impact of sample size on statistical inference enables researchers to make informed, resource-efficient decisions during study planning, and helps readers critically evaluate and appropriately interpret research findings.^8^ This study used data simulations to investigate how sample size affects statistical outcomes and its relationships with key metrics of statistical inference. In addition, two classical examples are provided to demonstrate sample size calculation methods for both continuous and binary outcomes.

## METHODS

This study used data simulations to examine the impact of sample size on key metrics of statistical inference, including type I error, type II error, statistical power, *p*-value, and 95% confidence interval. All statistical analyses were two-sided, with a significance level of *α* = 0.05. Analyses were conducted using R software, and figures were generated using the ggplot2 package.^9^

**Figure 1:**
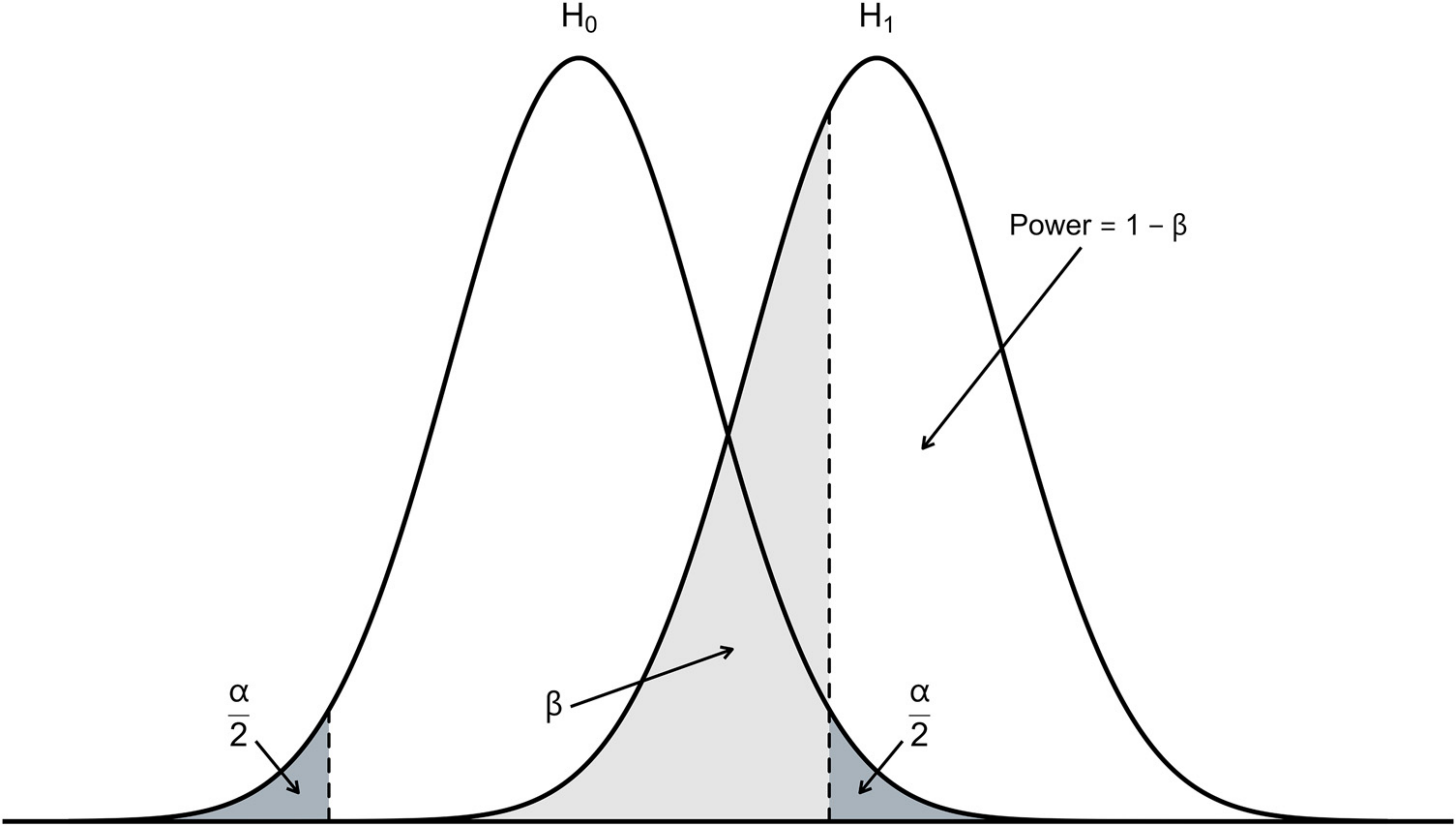
Relationships between type I error, type II error, and statistical power. *H_0_*: null distribution; *H_1_*: alternative distribution; *α*: significance level (set at 0.05), representing the probability of a type I error; and *β*: probability of a type II error. The two dashed lines indicate the rejection boundaries under *H_0_*. Shifting the boundaries toward the tails (e.g., decreasing *α* from 0.05 to 0.01) reduces the probability of a type I error (dark gray areas under *H_0_*) but increases the probability of a type II error (light gray area under *H_1_*).

The relationship between type I and type II errors was illustrated by simulating two normal distributions, representing the null (*H_0_*) and alternative (*H_1_*) hypotheses. The two distributions were assumed to have different means (*µ_0_* = 0, *µ_1_* = 3.5) but the same standard deviation (*σ* = 1.5). For a two-sided test with *α* = 0.05, the critical values were set at the 2.5th and 97.5th percentiles of the null distribution. The areas corresponding to type I error (*α*), type II error (*β*), and statistical power (1 – *β*) were labeled to depict their probabilities and interrelationships (Figure 1).^10^

**Figure 2:**
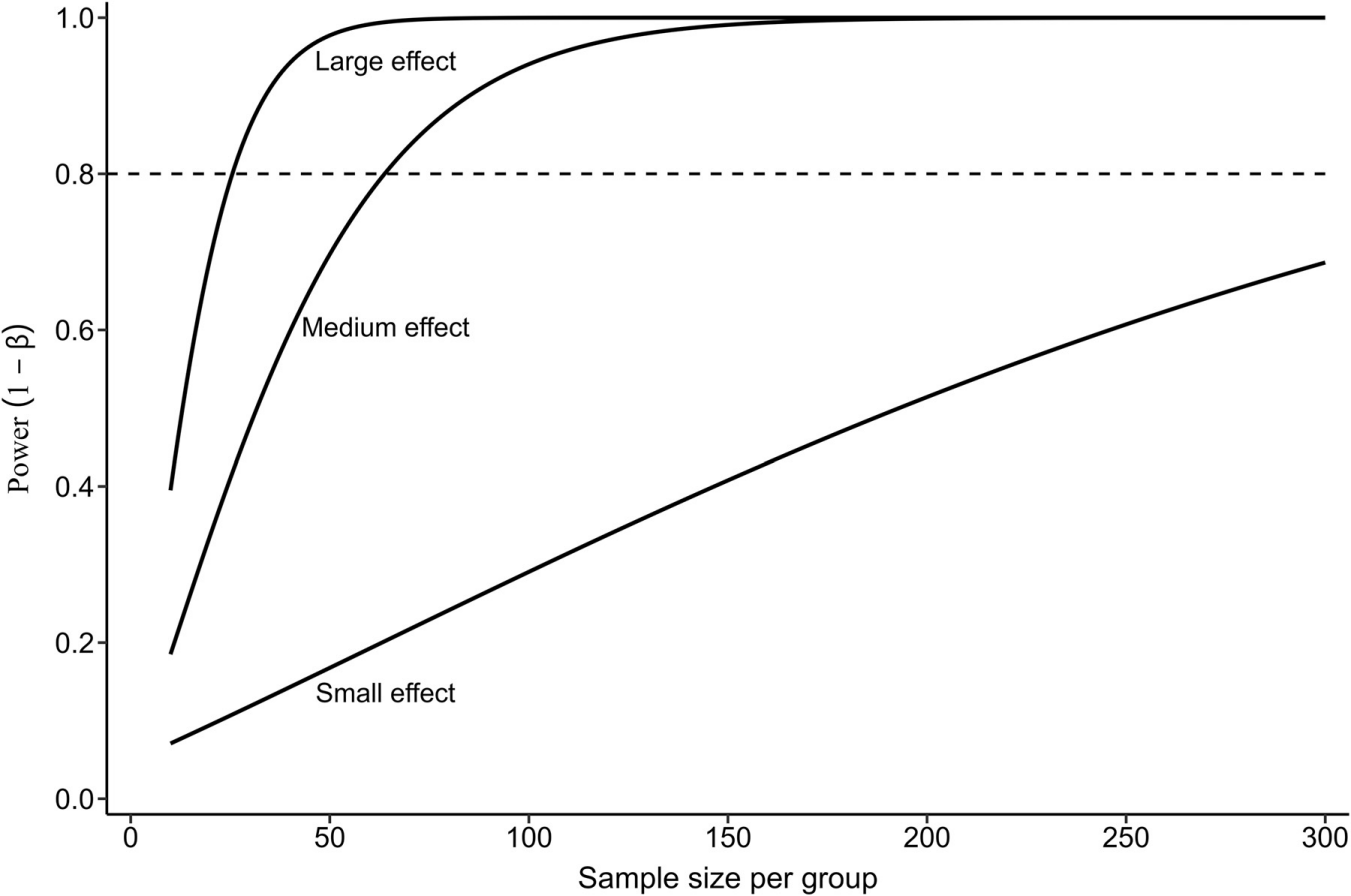
Relationship between sample size and statistical power, stratified by effect size. Statistical power was calculated using a two-sample, two-sided *t*-test with equal sample sizes from 10 to 300 per group and Cohen's effect sizes of small (*d* = 0.2), medium (*d* = 0.5), and large (*d* = 0.8) at *α* = 0.05. The dashed line indicates a power of 0.80.

To demonstrate how statistical power varies with sample size and effect size, power estimates were calculated using the two-sample *t*-test for equal sample sizes ranging from 10 to 300 per group. Cohen’s standardized effect sizes (*d*) of 0.2, 0.5, and 0.8 were used to represent small, medium, and large effects, respectively.^11^ Power calculations were performed using the pwr.t.test() function from the pwr package in R,^12^ assuming normality and equal variances between groups (Figure 2).

**Figure 3:**
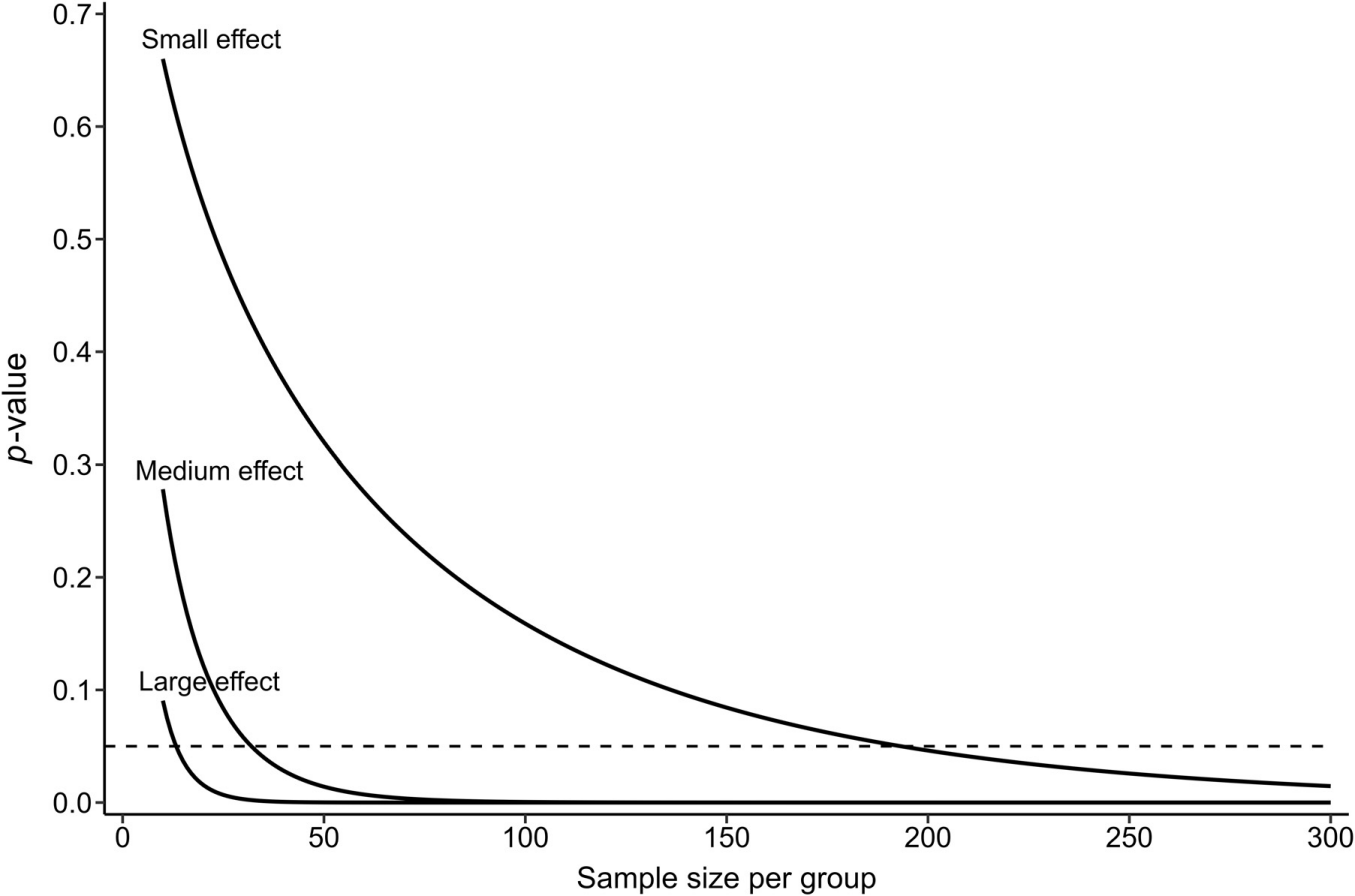
Relationship between sample size and *p*-values, stratified by effect size. *P*-values were calculated using a two-sample, two-sided *t*-test with equal sample sizes from 10 to 300 per group and Cohen's effect sizes of small (*d* = 0.2), medium (*d* = 0.5), and large (*d* = 0.8) at *α* = 0.05. The dashed line indicates *p* = 0.05.

**Figure 4:**
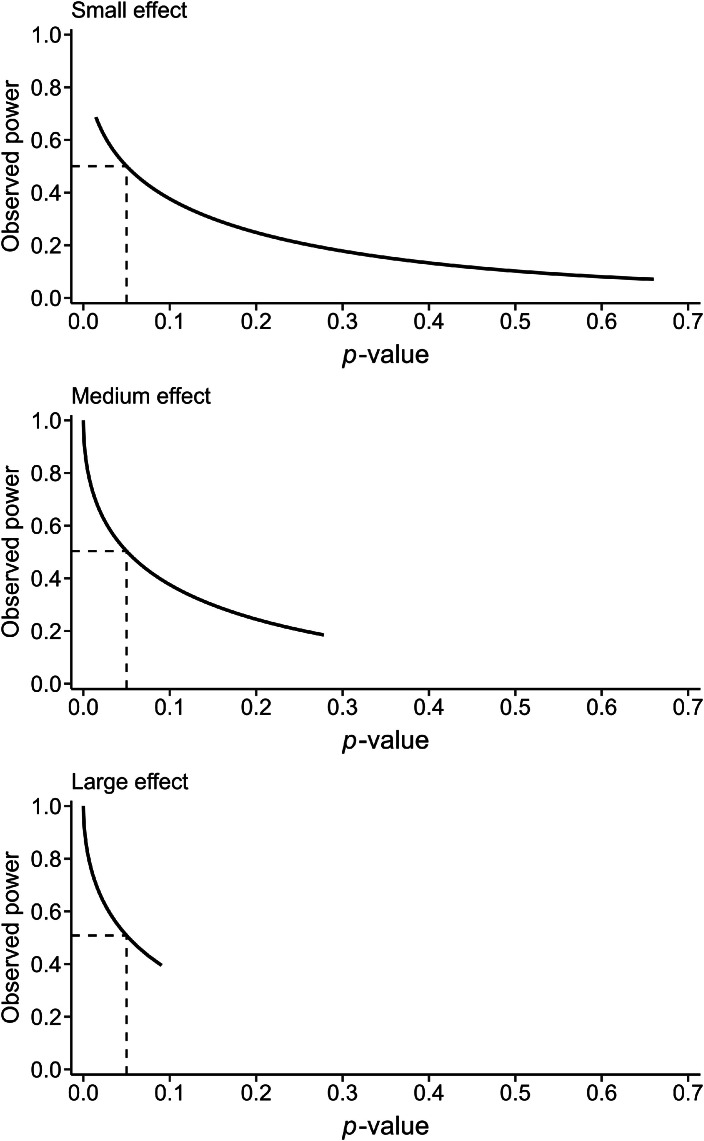
Relationship between *p-*values and observed power, stratified by effect size. *P*-values and observed power were calculated using a two-sample, two-sided *t*-test with equal sample sizes from 10 to 300 per group and Cohen's effect sizes of small (*d* = 0.2), medium (*d* = 0.5), and large (*d* = 0.8) at *α* = 0.05. In each panel, the vertical dashed line indicates *p* = 0.05, and the horizontal dashed line indicates the corresponding observed power.

The relationships of *p*-values with sample size and effect size were examined through a theoretical analysis of the two-sample *t*-test. The analysis considered equal sample sizes (*n*) ranging from 10 to 300 per group and Cohen’s standardized effect sizes (*d*) of 0.2, 0.5, and 0.8, assuming normality and equal variances between groups. For each combination of *n* and *d*, the noncentrality parameter $\delta =d\sqrt{\left(n/2\right)}$ was calculated under the alternative hypothesis, representing the expected *t*-statistic. The corresponding theoretical *p*-value was calculated from the central *t*-distribution with 2*n* – 2 degrees of freedom, assuming the null hypothesis is true. This analysis illustrates how *p*-values decrease as sample size increases for a given effect size (Figure 3). Furthermore, for each combination of *n* and *d*, observed (post hoc) power was calculated using the same method described for Figure 2. The resulting powers were plotted against their corresponding *p*-values to illustrate how observed power varies with *p*-values across different sample sizes and effect sizes (Figure 4).

**Figure 5:**
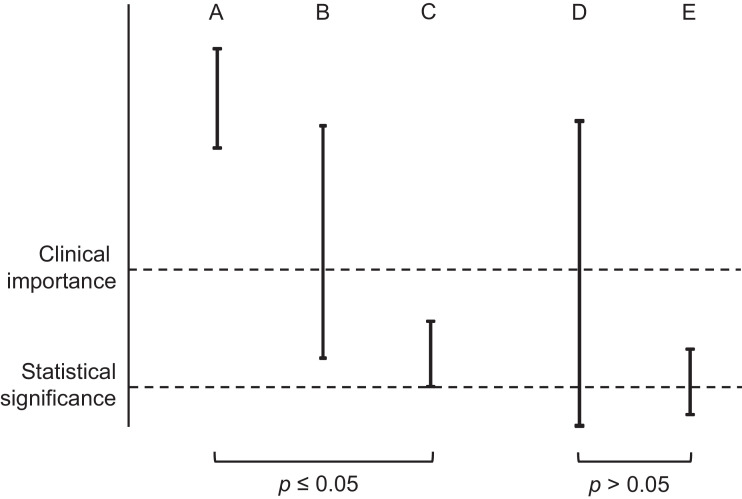
95% confidence intervals in relation to statistical significance and clinical importance. A: The effect estimate is statistically significant and clinically important. B: The effect estimate is statistically significant, but its clinical importance is uncertain due to the wide confidence interval. C: The effect estimate is statistically significant (*p* = 0.05) but not clinically important. D: The wide confidence interval indicates low precision and insufficient statistical power, making the effect estimate unreliable. E: The effect estimate is neither statistically significant nor clinically important.

To illustrate the distinct insights provided by *p*-values and 95% confidence intervals, five representative scenarios were constructed using hypothetical data.^13^^,^ ^14^ These scenarios demonstrate how 95% confidence intervals can be interpreted to assess whether an observed effect is statistically significant, clinically important, both, or neither. They also highlight that the width of a confidence interval, which is mainly determined by sample size, reflects the precision and uncertainty of effect estimates (Figure 5).

## RESULTS AND DISCUSSION

### Basic Concepts: Type I Error, Type II Error, and Statistical Power

Hypothesis testing is conducted under the null hypothesis, which assumes that there is no difference in the population parameters between the groups being compared. A type I error, or false positive, occurs when the null hypothesis is true but is rejected based on the sample data. To control the probability of such errors, researchers specify a significance level (*α*), which represents the maximum acceptable probability of making a type I error (Figure 1). The significance level is conventionally set at 0.05, meaning that if the null hypothesis is true, each individual test has a 5% probability of incorrectly rejecting the null hypothesis. In repeated independent studies using random samples from the same target population, the observed proportion of such errors may vary due to random variation but will average approximately 5%.

A type II error, or false negative, occurs when the null hypothesis is false but is not rejected based on the sample data. The probability of a type II error, denoted by *β*, is conventionally set at 0.20 in sample size calculations (Figure 1). This means that when the null hypothesis is false, each individual test has a 20% probability of failing to reject the null hypothesis. Across repeated independent studies using random samples from the same target population, the observed proportion of such errors may vary, but on average it will be approximately 20%. The substantial difference in the conventional thresholds for type I error (*α* = 0.05) and type II error (*β* = 0.20) reflects a greater emphasis on minimizing false positives than false negatives.

Another key metric closely related to type II error is statistical power (Figure 1), which is defined as the probability of correctly rejecting the null hypothesis (1 – *β*) when a true effect is present. Statistical power reflects a study’s ability to detect a true effect; it is determined by effect size, variability, sample size, and significance level. In sample size calculations, *α* is typically set at 0.05 and *β* at 0.20, resulting in a power (1 – β) of 0.80. This means that if the effect of interest is truly present, the study has an 80% probability of detecting it, given the specified sample size and significance level.

### Relationships between Type I Error, Type II Error, and Sample Size

In hypothesis testing based on sample data, either a type I or type II error may occur. For a fixed sample size, the probability of a type I error can be reduced by adopting a more stringent significance level (e.g., *α* = 0.01 instead of 0.05); however, this adjustment will increase the probability of a type II error, and vice versa (Figure 1). For a fixed significance level (*α*), as sample size increases, the standard error ($\sigma /\sqrt{n})\;$decreases, while the means remain unchanged. This causes both the null and alternative distributions to narrow, reducing their overlap. Consequently, the probability of a type II error (the light gray area under *H_1_*) decreases, and statistical power (1 − *β*) increases (Figure 1).

The probability of a type I error is determined solely by the significance level (*α*) and is not affected by effect size or sample size. Therefore, the probability of a type I error is the same for all hypothesis tests that reject the null hypothesis at the same significance level (e.g., *α* = 0.05). However, the actual strength of evidence against the null hypothesis, as reflected by the observed *p*-values, can differ substantially. For example, a *p*-value of 0.001 provides much stronger evidence against the null hypothesis than a *p*-value near 0.05, even though both result in rejection of the null hypothesis at the 0.05 significance level.

When type I error is the primary concern, as in clinical trials evaluating medication efficacy and safety, a more stringent significance level (e.g., *α* = 0.01) is often used to minimize the risk of false positives. Conversely, when type II error is the greater concern, a more lenient significance level may be adopted. For example, heterogeneity tests in meta-analyses use *α* = 0.10 rather than 0.05 to increase the probability of detecting heterogeneity among the included studies (Figure 1).

When a hypothesis test fails to reject the null hypothesis, there are two possible explanations: either the null hypothesis is true, or it is false but was not rejected (a type II error) due to factors such as high variability or insufficient sample size. Because the presence of a true effect cannot be known with certainty, statistical evidence alone cannot distinguish between these two scenarios.

### Relationships of Statistical Power with Effect Size and Sample Size

Figure 2 demonstrates that statistical power depends strongly on both effect size and sample size. For a fixed effect size, statistical power increases with larger sample size and gradually levels off as it approaches the maximum, where further increases in sample size have little influence on statistical power. For a fixed sample size, smaller effect sizes yield lower power. To achieve desired power, such as 0.80, smaller effect sizes require larger sample sizes (Figure 2).

For a given study, the most effective way to reduce type II error (and increase statistical power) is to increase the sample size, especially when the effect size is small (Figure 2). When a hypothesis test fails to reject the null hypothesis, the possibility of a type II error may be a concern. However, post hoc power analysis provides little meaningful insight and can be misleading, as the calculated power is typically low when the observed *p*-value exceeds the significance level (Figure 4). This is because post hoc power is mathematically determined by the observed *p*-value, sample size, and significance level, and thus provides little additional information beyond that contained in the *p*-value.^15^^,^ ^16^ Power analysis should be conducted *a priori* during study planning to ensure adequate statistical power and enable confident interpretation of non-significant results.

### Relationships of *p*-value with Effect Size and Sample Size

A *p*-value is the probability of obtaining a test statistic at least as extreme as the one observed in the current sample, assuming that the null hypothesis is true. The observed *p*-value is compared with the significance level (*α*) to determine whether the null hypothesis should be rejected. If the *p*-value is less than or equal to *α*, the null hypothesis is rejected, with smaller *p*-values indicating stronger evidence against the null hypothesis and in favor of the alternative hypothesis. It should be noted that, in frequentist analysis, the *p*-value does not represent the probability that the null hypothesis is true, as it may be interpreted in a Bayesian framework.^17^

Figure 3 demonstrates that *p*-values are highly dependent on both effect size and sample size. For a fixed effect size, *p*-values decrease rapidly as sample size increases, then gradually level off near the minimum, where further increases in sample size have little influence on the *p*-values. For a fixed sample size, smaller effect sizes yield larger *p*-values. To achieve a specific *p*-value, such as 0.05, smaller effect sizes require larger sample sizes. It is important to recognize that smaller *p*-values do not necessarily indicate larger effect sizes, they may simply reflect larger sample sizes (Figure 3).

### *P*-value and 95% Confidence Interval

Hypothesis tests generate *p*-values, effect estimates, and confidence intervals to evaluate differences in population parameters between groups. A *p*-value quantifies the compatibility of the observed data with the null hypothesis and is used to assess statistical significance. A confidence interval represents a range of plausible values for the true effect size, based on the sample data and a specified confidence level. It reflects not only the direction and magnitude of the effect, but also the precision and uncertainty of the estimate due to sampling variability. At a 0.05 significance level, the corresponding 95% confidence interval means that if the study were repeated many times using random samples from the same target population, approximately 95% of the resulting confidence intervals would contain the true effect.

Both *p*-values and 95% confidence intervals are commonly used in statistical inference; however, 95% confidence intervals provide a more complete picture by conveying statistical significance, precision and uncertainty, and clinical importance.

First, a 95% confidence interval provides valuable insight into the corresponding *p*-value. If the interval does not include the null value (e.g., 0 for a mean difference, 1 for a relative risk or odds ratio), the *p*-value is less than 0.05 (Figure 5A, 5B). When the null value lies exactly at the lower or upper bound of the interval, the *p*-value is approximately 0.05 (Figure 5C). If the null value falls within the interval, the *p*-value is greater than 0.05 (Figure 5D, 5E).

Second, the width of a 95% confidence interval reflects the precision and uncertainty of the effect estimate, and it is inversely related to statistical power. A wider confidence interval indicates lower precision and insufficient statistical power (Figure 5B, 5D), which is particularly concerning when the interval includes the null value (Figure 5D). In such cases, it is not possible to draw reliable conclusions. Increasing the sample size can improve the precision of effect estimates by narrowing the confidence intervals, and can also increase statistical power when true effects are present.

Finally, 95% confidence intervals can help determine whether the observed effect is clinically important (Figure 5A, 5C, 5E). In contrast, a *p*-value less than 0.05 does not necessarily indicate clinical importance (Figure 5B, 5C), since a small *p*-value may result from a large sample size even when the effect size is clinically negligible (Figure 3).

Because confidence intervals provide more comprehensive information, it is important to report both effect estimates and confidence intervals alongside *p*-values to facilitate transparent and meaningful interpretation of research findings.^13^

### Examples of Sample Size Calculation

In medical research, the two-sample *t*-test and two-sample proportion *z*-test are commonly used to compare means or proportions between two independent groups. This section demonstrates sample size calculations for these tests using the pwr package in R.^12^

Example 1 **Two-sample *t*-test to compare group means**

The required sample size per group for a two-sample *t*-test can be calculated using the formula:^10^

$$n\;=\;{\left(Z_{1-a/2}\;+\;Z_{1-\beta }\right)}^{2}\;\times \;\frac{\sigma_{1}^{2}+\sigma_{2}^{2}}{{\left(\mu_{1}-\mu_{2}\right)}^{2}}$$

Where *n* is the required sample size per group; *Z_1−α/2_* and *Z_1−β_* are the critical values from the standard normal distribution corresponding to the two-sided significance level (*α*) and the desired statistical power (1 – *β*), respectively; *σ_1_* and *σ_2_* are the population standard deviations (SD) for groups 1 and 2; and *µ_1_* and *µ_2_* are the population means for groups 1 and 2.

This formula is based on the normal approximation, which uses the standard normal (*Z*) distribution rather than the exact *t*-distribution. This normal approximation is appropriate when the sample size is large (e.g., *n* ≥ 30 per group), as the central limit theorem ensures that the sampling distribution of the mean difference is approximately normal. For smaller sample sizes (e.g., *n* < 30), the *t*-distribution should be used to account for the additional variability in estimating the standard error.

Assuming equal variances, and using sample data to estimate the population parameters, the sample size formula can be simplified as:

$$n\;=\;2\times {\left(Z_{1-a/2}+\;Z_{1-\beta }\right)}^{2}\;\times \;{\left(\frac{s_{p}}{{\bar{x}}_{1}-\;{\bar{x}}_{2}}\right)}^{2}$$

$$s_{p}=\;\sqrt{\frac{\left(n_{1}-1\right)s_{1}^{2}\;+\;\left(n_{2}-1\right)s_{2}^{2}}{n_{1}\;+\;n_{2}\;-\;2}}$$

Here *s_p_* is the pooled SD from the two samples; ${\bar{x}}_{1}$ and ${\bar{x}}_{2}$ are the sample means; $n_{1}$ and $n_{2}$ are the sample sizes; and $s_{1}$ and $s_{2}$ are the sample SDs. In practice, sample means and SDs are typically estimated from pilot studies or prior literature.

This formula shows that in addition to the significance level (*α*) and statistical power (1 – *β*), the difference between group means (${\bar{x}}_{1}-\;{\bar{x}}_{2}$) and variability (*s_p_*) are key determinants of the required sample size. Cohen’s effect size (*d*), also known as the standardized mean difference, quantifies the magnitude of the difference between two means while accounting for variability:^11^

$$d\;=\;\frac{{\bar{x}}_{1}-{\bar{x}}_{2}}{s_{p}}$$

The required sample size per group for a two-sample *t*-test can be calculated using the pwr package in R:^12^

pwr.t.test(n=NULL,d=NULL,sig.level=NULL,power=NULL,type="two.sample",alternative="two.sided")

In this function, pwr.t.test specifies a power analysis for a *t*-test, and NULL indicates that the parameter value will be provided or calculated. The parameters include: *n,* the required sample size for each group; *d,* Cohen’s effect size; sig.level, the significance level (e.g., 0.05); power, the desired statistical power (e.g., 0.80); type, which specifies a two-sample test; and alternative, which specifies a two-sided test.

The pwr.t.test() function includes four key parameters: sample size per group (*n*), Cohen’s effect size (*d*), significance level (*α*), and statistical power (1 – *β*). Any one of these parameters can be calculated when the other three are specified. For example, by setting *d* = 1.1, sig.level = 0.05, power = 0.80, and *n* = NULL, the function estimates that approximately 15 subjects per group are required.

In addition to the coding-based approach, G*Power is a widely used software tool that provides an interactive, menu-driven interface for conducting power and sample size calculations.^18^

If information on the sample means and standard deviations is unavailable, Cohen recommends using conventional standardized effect sizes (Cohen's *d*) to approximate required sample sizes: 0.2 for a small effect, 0.5 for a medium effect, and 0.8 for a large effect.^11^ Assuming *α* = 0.05 and power = 0.80 for a two-sample, two-sided *t*-test, the required sample sizes per group to detect small, medium, and large effect sizes are approximately 394, 64, and 26 subjects, respectively.

Example 2 **Two-sample *z*-test to compare group proportions**

The required sample size per group for a two-sample proportion *z*-test can be calculated as:^10^

$$n\;=\;{\left(Z_{1-a/2}\;+\;Z_{1-\beta }\right)}^{2}\;\times \;\frac{p_{1}\left(1-p_{1}\right)\;+\;p_{2}\left(1-p_{2}\right)\;}{{\left(p_{1}\;-\;p_{2}\right)}^{2}}$$

Where *n* is the required sample size per group; *Z_1-α/2_* and *Z_1-β_* are the critical values from the standard normal distribution corresponding to the two-sided significance level (*α*) and the desired statistical power (1 − *β*), respectively; and *p_1_* and *p_2_* are the expected population proportions in the two groups.

This formula relies on the normal approximation to the binomial distribution, which is appropriate when the expected counts of events and non-events satisfy np ≥ 5 and n(1 − p) ≥ 5. For small samples or extreme proportions, exact methods such as Fisher’s exact test or the exact binomial test provide more accurate results.

Cohen’s effect size (*h*), also known as the standardized difference in proportions, quantifies the magnitude of the difference between two proportions while accounting for variability using an arcsine square root transformation:^11^

$$h\;=\;2\arcsin\sqrt{p_{1}}-2arcsin\sqrt{p_{2}}$$

The required sample size per group for a two-sample proportion *z*-test can be calculated using the pwr package in R:^12^

pwr.2p.test(n=NULL,h=NULL,sig.level=NULL,power=NULL,alternative="two.sided")

$$h=ES.h\left(p_{1},\;p_{2}\right)$$

In this function, pwr.2p.test specifies a power analysis for a two-sample proportion *z*-test. NULL indicates that the parameter value will be provided or calculated. The parameters include: *n,* the required sample size per group; *h,* Cohen's effect size, calculated using the ES.h function; *p_1_* and *p_2_*, the proportions for groups 1 and 2; sig.level, the significance level (e.g., 0.05); power, the desired statistical power (e.g., 0.80); and alternative, which specifies a two-sided test.

As with the two-sample *t*-test, the pwr.2p.test() function includes four parameters: sample size per group (*n*), Cohen’s effect size (*h*), significance level (*α*), and statistical power (1 – *β*). Any one of these parameters can be calculated when the other three are specified. For example, if the proportion of subjects experiencing an event is 30% in group 1 (*p_1_* = 0.3) and 50% in group 2 (*p_2_* = 0.5), Cohen’s effect size (*h* = -0.41) can be calculated using ES.h (*p_1_* = 0.3, *p_2_* = 0.5). Given sig.level = 0.05, power = 0.80, and *n* = NULL, the function estimates that the required sample size is approximately 93 subjects per group. Alternatively, the same result can be obtained using G*Power software.^18^

### Simulation-Based Power and Sample Size Analysis

When standard formulas or software are not applicable, such as in mixed-effects models or when model assumptions are violated, simulation-based power analysis provides a flexible and reliable approach for estimating statistical power and determining required sample size.^7^^,^ ^19^^,^ ^20^ This approach begins by generating a simulated dataset based on anticipated exposure parameters (e.g., mean and standard deviation), expected effect size for a health outcome (e.g., relative risk), and a proposed sample size. The planned statistical analysis (e.g., a linear mixed-effects model for panel studies^21^) is then applied to the simulated dataset to assess the exposure-outcome relationship at a specified significance level (e.g., *α* = 0.05). The resulting *p*-value is used to determine whether the null hypothesis is rejected. This process is repeated many times (e.g., more than 1,000 iterations), the proportion of simulations that yield statistically significant results (*p*-value ≤ *α*) represents the statistical power for the given sample size. Once the statistical model is established, a power curve can be generated to show how power changes with sample size.^20^ This curve can be used to identify the minimum sample size required to achieve desired statistical power. Simulation-based methods are widely applicable for estimating sample size across various statistical analyses.

### Limitations of Sample Size Estimation

Sample size estimation methods have inherent limitations that should be considered. A key challenge is the reliance on assumed effect sizes, such as mean differences and standard deviations, which are unknown during study planning. These effect estimates are typically derived from pilot studies or prior literature but often carry substantial uncertainty. Even a modest overestimation of the effect size can result in underpowered studies, increasing the risk of false-negative results. Conversely, underestimating the effect size may lead to unnecessarily large and resource-intensive studies that detect statistically significant but clinically negligible effects. Both scenarios can compromise statistical validity and limit meaningful interpretation of research findings.

Furthermore, sample size formulas are typically based on simplified assumptions, such as normality, independence, and equal variances, and may not account for real-world complexities, including loss to follow-up, treatment nonadherence, missing or clustered data, and the need for multivariable adjustment or subgroup analyses. These factors can reduce effective sample size and diminish statistical power. Therefore, incorporating realistic assumptions into sample size estimation process, such as accounting for a 10% attrition rate in clinical trials, is necessary to ensure adequate power under practical conditions.

Finally, sample size estimation cannot eliminate the inherent uncertainty in statistical inference, which may lead to misinterpretation of study findings. Even well-powered studies (e.g., power ≥ 80%) may yield non-significant results due to: (1) the null hypothesis being true (i.e., no real effect); (2) a type II error resulting from sampling variability, since 80% power implies a 20% chance of failing to detect a true effect; or (3) overestimation of the effect size during study design, leading to insufficient power and an increased risk of type II error. Conversely, underpowered studies (e.g., power < 80%) may still produce statistically significant results due to: (1) the presence of a true effect, since even underpowered studies retain some ability to detect it; (2) a type I error arising from sampling variability, as a 0.05 significance level implies a 5% chance of incorrectly rejecting the null hypothesis; or (3) an observed effect size larger than anticipated, resulting in greater power and a reduced risk of type II error. In all cases, statistical significance or nonsignificance alone cannot confirm or refute the presence of a true effect.^17^ This limitation underscores the importance of interpreting *p*-values in conjunction with effect estimates, confidence intervals, and other relevant scientific evidence.

## CONCLUSIONS

Sample size estimation is a critical component of rigorous study design, aimed at determining an appropriate number of subjects based on expected effect size, variability, significance level, and desired statistical power. Adequately powered studies are more likely to detect true effects, produce reliable and precise effect estimates, and enable confident interpretation of non-significant results. In contrast, underpowered studies may fail to detect true effects, resulting in non-significant *p*-values, wide confidence intervals, and inconclusive findings. In such cases, a non-significant result may suggest insufficient power rather than the absence of a true effect. Because observed power is mathematically linked to the *p*-value, post hoc power analysis provides little additional value, as it merely reiterates information already conveyed by the *p*-value and confidence interval.

*P*-values are influenced by both effect size and sample size. For a fixed effect size, *p*-values decrease as sample size increases. Therefore, a smaller *p*-value does not necessarily indicate a larger effect size; it may simply reflect a larger sample size. In contrast, as sample size increases, effect estimates remain relatively stable, while their 95% confidence intervals become narrower, reflecting improved precision. Compared with *p*-values, 95% confidence intervals provide more comprehensive information, including direction, magnitude, precision, statistical significance, and clinical importance of effect estimates. For this reason, reporting effect estimates and 95% confidence intervals alongside *p*-values is essential to facilitate transparent and meaningful interpretation of research findings.
